# The activation of the oxidative stress response transcription factor SKN-1 in *Caenorhabditis elegans* by mitis group streptococci

**DOI:** 10.1371/journal.pone.0202233

**Published:** 2018-08-16

**Authors:** Ali Naji, John Houston IV, Caroline Skalley Rog, Ali Al Hatem, Saba Rizvi, Ransome van der Hoeven

**Affiliations:** Department of Diagnostic and Biomedical Sciences, School of Dentistry, University of Texas Health Science Center, Houston, Texas, United States of America; Texas A&M University Health Sciences Center, UNITED STATES

## Abstract

The mitis group, a member of the genetically diverse viridans group streptococci, predominately colonizes the human oropharynx. This group has been shown to cause a wide range of infectious complications in humans, including bacteremia in patients with neutropenia, orbital cellulitis and infective endocarditis. Hydrogen peroxide (H_2_O_2_) has been identified as a virulence factor produced by this group of streptococci. More importantly, it has been shown that *Streptococcus oralis* and *S*. *mitis* induce epithelial cell and macrophage death via the production of H_2_O_2_. Previously, H_2_O_2_ mediated killing was observed in the nematode *Caenorhabditis elegans* in response to *S*. *oralis* and *S*. *mitis*. The genetically tractable model organism *C*. *elegans* is an excellent system to study mechanisms of pathogenicity and stress responses. Using this model, we observed rapid H_2_O_2_ mediated killing of the worms by *S*. *gordonii* in addition to *S*. *mitis* and *S*. *oralis*. Furthermore, we observed colonization of the intestine of the worms when exposed to *S*. *gordonii* suggesting the involvement of an infection-like process. In response to the H_2_O_2_ produced by the mitis group, we demonstrate the oxidative stress response is activated in the worms. The oxidative stress response transcription factor SKN-1 is required for the survival of the worms and provides protection against H_2_O_2_ produced by *S*. *gordonii*. We show during infection, H_2_O_2_ is required for the activation of SKN-1 and is mediated via the p38-MAPK pathway. The activation of the p38 signaling pathway in the presence of *S*. *gordonii* is not mediated by the endoplasmic reticulum (ER) transmembrane protein kinase IRE-1. However, IRE-1 is required for the survival of worms in response to *S*. *gordonii*. These finding suggests a parallel pathway senses H_2_O_2_ produced by the mitis group and activates the phosphorylation of p38. Additionally, the unfolded protein response plays an important role during infection.

## Introduction

Members of the mitis group of streptococci, which are also part of the viridans group of streptococci (VGS), are commensals of the oral cavity and the upper respiratory tract of humans [1]. This group of microorganisms includes *Streptococcus gordonii*, *S*. *mitis*, *S*. *oralis*, *S*. *sanguinis*, and a few related species. Mitis group streptococci have been recognized as causative agents of a variety of human diseases such as infective endocarditis, orbital cellulitis and more recently increased incidences of bacteremia in patients with neutropenia [2–5]. More importantly unique strains of *S*. *mitis* and *S*. *oralis* that cause bacteremia have been isolated from cancer patients that have undergone chemotherapy [6, 7]. These invasive strains are resistant to more antimicrobial agents than other VGS species, and as a consequence increase the risk of adverse patient outcomes [8]. Despite the clinical significances of these infections, the mechanisms of pathogenesis and the pathophysiology are poorly understood. In addition, due to increased antibiotic resistance, novel therapeutic strategies are required to combat these organisms.

The mitis group is known to produce hydrogen peroxide (H_2_O_2_) which has been shown to play important roles in oral microbial communities [9]. H_2_O_2_ produced by *S*. *gordonii* and *S*. *sanguinis* has been reported to reduce the growth of cariogenic *S*. *mutans* and several other pathogenic bacteria [10, 11]. In addition, H_2_O_2_ stimulates the release of DNA which is important for biofilm formation and enables the exchange of genetic material among bacteria [12]. However, recent studies have highlighted a role for H_2_O_2_ as a cytotoxin that induces macrophage, neutrophil and epithelial cell death [13–15]. A more recent study demonstrated H_2_O_2_ produced by *S*. *oralis* mediates damage of lysosomes resulting in macrophage death [16]. Furthermore, pneumococcal H_2_O_2_ induced the activation of stress related cellular signaling pathways confirming the role of H_2_O_2_ as an important virulence factor of the mitis group [17].

*C*. *elegans*, a microscopic nematode is an excellent model system for studying pathogenesis, immunity and oxidative stress. In its natural habitat, the worms consume bacteria as their food source and thereby encounter numerous threats and insults from the ingested microbes [18]. This has ensured a strong selection pressure to evolve and maintain an immune response within the intestinal cells of the worm that is capable of producing a targeted response [19]. In the worm the mechanisms underlying pathogenesis are mediated through an infection like process or by diffusible toxins. Previously, H_2_O_2_ produced by *Enterococcus faecium*, *S*. *pyogenes*, *S*. *pneumonia*, *S*. *oralis* and *S*. *mitis* was shown to be responsible for killing *C*. *elegans* [20–22].

Phase II reactions provide protection against Reactive Oxygen Species (ROS)-induced oxidative stress damage in tissues that are associated with many diseases and aging [23, 24]. Enzymes supporting these detoxification reactions are involved in the biosynthesis of glutathione (GCS-1) and its conjugation of substances such as glutathione-S-transferases (GSTs). The induction of phase II gene expression has been shown to be regulated by the Cap and Collar (CnC) transcription factors. For example Nrf-2 (erythroid-derived-2)-like-2) in mammals and SKN-1 in *C*. *elegans*, mainly control transcription of the phase II response [25, 26]. Several studies in *C*. *elegans* have shed light into the regulation of SKN-1 an orthologue of Nrf-2 [25, 27]. SKN-1 is activated in response to different stimuli mediated by several regulatory pathways [28–30]. Oxidative stress induced activation is facilitated by the phosphorylation of SKN-1 via the p38 MAPK pathway [31]. This signal pathway is comprised of the MAPKKK, NSY-1 (ASK-1 homologue), the MAPKK, SEK-1 and the MAPK, PMK-1 (p38 homologue). Similarly, ROS produced by the dual oxidase Ce-DUOX-1/BLI-3 in response to pathogens activates SKN-1 through the p38 MAPK pathway [32]. Until recently, the primary trigger that activates the p38 MAPK pathway by oxidative stress in the worms was not discovered. Work published by Hourihan et al demonstrated the endoplasmic reticulum (ER) transmembrane protein IRE-1 initiates the p38/SKN-1(Nrf2) antioxidant response by ROS that are generated at the ER or by the mitochondria [33].

In this study, we demonstrate members of the mitis group of streptococci are able to colonize and cause death of *C*. *elegans* via the production of H_2_O_2_. We demonstrate SKN-1 is required for the survival of the worms and is protective against these pathogens when overexpressed. Furthermore, the activation of SKN-1 is via the p38 MAPK pathway. However, based on our data the activation of p38 MAPK pathway does not require IRE-1 as previously seen during oxidative stress induced by arsenite. Moreover, in the presence of *S*. *gordonii* phosphorylation of PMK-1 is not dependent on Ce-Duox1/BLI-3.

## Materials and methods

### *C*. *elegans* strains

*C*. *elegans* strains were grown and maintained as previously described [34]. The strains used in this study are listed in the supplementary data (**S1 Table**).

### Bacterial strains

All streptococci strains were grown in Todd Hewitt media supplemented with 0.3% yeast extract (THY) with or without sheep blood, while *E*. *coli* strains were grown in Luria-Bertani (LB) broth in the presence or absence of antibiotics streptomycin or carbenicillin. Strains used in this study were S. *gordonii* DL1 Challis (WT), S. *gordonii Challis* DL1 Δ*spxB*, *S*. *gordonii Challis* DL1 ΔspxB;*spxB*^+^, *S*. *mitis* ATCC, *S*. *oralis* ATCC, *S*. *mutans*, *S*. *salivarius*, and *E*. *coli* OP50. Clinical isolates of *S*. *mitis* (VGS# 3 and 4) and *S*. *oralis* (VGS# 10 and 13) were used in this study.

### Killing assays

For streptococcus killing assays, bacterial strains grown in THY medium with or without sheep blood for 16 hours were seeded on THY plates in the presence or absence of 1000U of catalase or 100U of superoxide dismutase and incubated at 37°C for 24 hours in a candle jar. 60–90 L4 larvae were transferred to two replica plates and incubated at 25°C under aerobic conditions. Larvae were scored for survival at various points along the time course. The experiment was repeated 3 times.

### Colonization of worms

Overnight cultures of the bacteria were pelleted, resuspended in PBS containing 10mM 5-(and-6)-Carboxy-X-Rhodamine, Succinimidyl Ester (5(6)-ROX, SE) and incubated at room temperature (RT). Thereafter, concentrated stained cultures were plated on to THY plates and incubated in the dark at RT for 30 minutes. L4 larvae were subsequently added to the plates and incubated at 25°C in the dark for 2 hours. Worms were collected using M9, anesthetized in M9 containing 0.1% sodium azide, mounted on 2% agarose pads and imaged using a Nikon C2 plus confocal microscope.

Worms exposed to the bacteria were collected and washed 3 times in M9 containing 25 mM levamisole. Thereafter, worms were exposed to 25 mM levamisole, 1mg/ml ampicillin and 1mg/ml streptomycin in M9 for 1 hour to kill the external bacteria. Worms were washed again in M9 containing 25 mM levamisole and five worms were disrupted in M9 using a motorized pestle. Serial dilutions of the lysate were grown overnight on THY at 37°C under microaerophilic conditions and the colony forming units were counted. At least three replicates for each strain of bacteria were processed.

### RNA Isolation and sequencing (RNAseq)

L4 larvae were exposed to bacterial strains for 2–3 hours. Thereafter, RNA was extracted from the worms using Trizol (Invitrogen) as recommended by the manufacturer. Subsequently, samples were treated with Tubro DNase I (Ambion) to remove DNA contamination according to manufacturer’s instructions. RNA samples were sequenced by Genewiz Inc. The average reads/sample was 35,106,625.25, while the average percentage of the bases with ≥Q30 reads was 98.4. Using CLC Genomics Server program the sequences were mapped to the *C*. *elegans* genome. After quantile normalization on RKPM values, unsupervised hierarchical clustering and Principal Component Analysis (PCA) were performed. The gene expression comparison between the control samples and the test samples was conducted using proportion based Kal’s test, which generalizes the RPKM as counts for 1 vs 1 comparison. For all the statistical tests, p-values and fold-change values were calculated. A list of differentially expressed genes with a normalized RPKM fold difference greater than 2 or less than -2 that had a FDR corrected p-value < 0.05 were selected as differentially expressed genes.

### Quantitative Real Time Polymerase Chain Reaction (qRT-PCR) Analysis

RNA was isolated as described above and qRT-PCR was performed on a Bio-rad CFX96 Touch Real-Time PCR Detection System using an iTaq Universal SYBR Green one-step kit (Bio-rad). Comparative C_T_ method was used to determine fold changes in gene expression normalized to *act-1*. Three biological replicates were performed. Primers used in this study were previously described [32].

### RNA Interference

RNAi was induced by feeding L1 worms through L4 stage with bacteria producing dsRNA to target genes. To induce *bli-3* knockdown in the worms, *E*. *coli* HT115(DE3) expressing *bli-3* RNAi was diluted in a 1:30 ratio with *E*.*coli* harboring the vector control as previously described [32]. RNAi expressing clones were obtained from the *C*. *elegans* library (Source Bioscience). All clones were verified by sequencing. Clones absent in the library were constructed as previously described [32].

### Fluorescence microscopy

To investigate the expression of *gcs-1*::*gfp*, worms were exposed to bacterial strains for 2 hours at 25°C, subsequently anesthetized using 0.1% sodium azide, mounted on 2% agarose pads, and imaged using a Nikon C2 plus confocal microscope. The levels of GFP expression were scored as previously described [32]. SKN-1B/C::GFP expression was analyzed by fluorescence microscopy in worms exposed to bacterial strains for 2–3 hours. Imaging was performed using the FITC and DAPI channels. Percentages of worms indicating the degree of nuclear localization of SKN-1B/C::GFP in the intestinal cells were scored as previously described [31]. All experiments were repeated more than three times.

### Western blot

Worms exposed to the bacterial strains for 2 hours were washed and collected in a 100 μl pellet using protein extraction buffer containing a protease inhibitor cocktail (Pierce) and a phosphatase inhibitor (Pierce). Pellets were sonicated two times on ice for 10 seconds at level 5 and 35 duty. The resulting suspensions were incubated with 1% SDS for 5 minutes and thereafter centrifuged at 14,000 XG for 10 minutes at 4°C. Supernatants were transferred to fresh Eppendorf tubes and the total protein concentration was estimated using a Bradford assay. Sample buffer was added to protein lysates and boiled for 5 minutes. For phospho-p38 detection, 20μg of total protein for each sample was resolved on a 12% SDS-PAGE gel and subsequently transferred to a PVDF membranes for 45 minutes at Room Temperature (RT) using a semidry transfer apparatus. The membranes were blocked with TBS-T blocking buffer for 1 hour. Thereafter, the membranes were incubated with 1:1000 Phospho-p38 antibodies (cell signaling) overnight at 4°C. The blots were washed 3 times with TBS-T and incubated with 1:5000 secondary antibodies conjugated to horseradish peroxidase for 1 hour at RT. Subsequently, the membranes were washed with TBS-T 3 times, incubated with enhanced chemiluminescent substrate (Pierce) for detection of horseradish peroxidase (HRP) activity for 1 minute and visualized using the Biorad Chemidoc Imaging System. The detection of α-tubulin by anti-α-tubulin antibodies was used as a loading control.

### Statistical analysis

All statistical analysis was performed using GraphPad Prism version 6.0 (GraphPad Software, San Diego, CA). Statistical differences for scored fluorescent micrographs were determined by Chi square and Fisher's exact tests. Each experimental condition was compared to the control condition. P-values of <0.05 were considered to be statistically significant. Statistically significant differences are indicated in the figures with asterisks next to the experimental condition. Kaplan-Meier log rank analysis was used to compare survival curves and to calculate the median survival. P-values <0.05 were considered to be statistically significant.

## Results and discussion

### Killing of the worms was mediated via H_2_O_2_ production by the mitis group of streptococci

**Fig 1:**
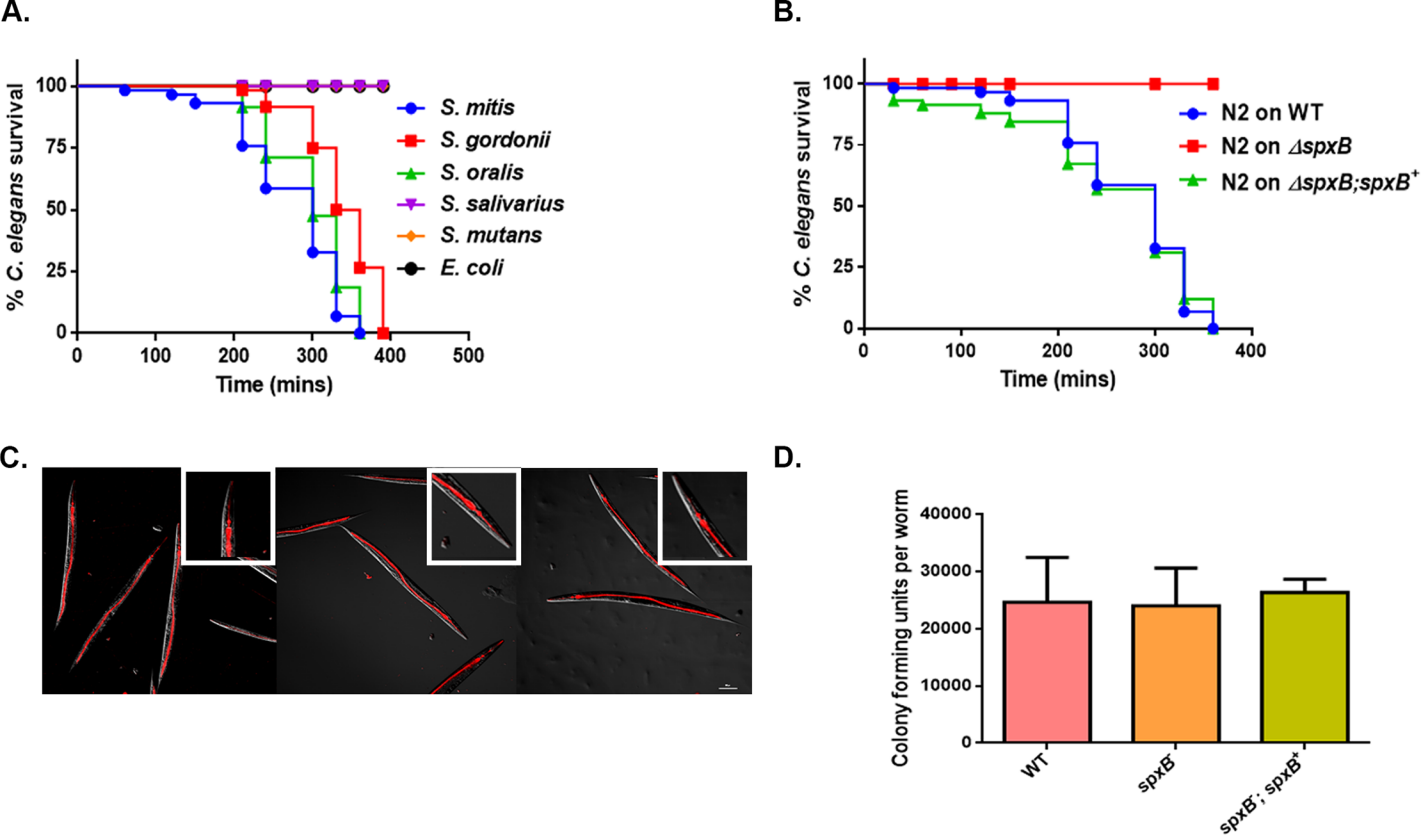
H_2_O_2_ mediated killing of *C*. *elegans* by mitis group streptococci. **(A)** Survival of N2 L4 larvae exposed to *S*. *gordonii*, *S*. *oralis*, *S*. *mitis*, *S*. *salivarius S*. *mutans* and *E*. *coli* OP50 on THY plates. **(B)** Survival of *S*. *gordonii* WT, Δ*spxB* mutant and Δ*spxB;spxB*^*+*^ complemented strains on N2 L4 larvae containing THY plates. The data are representative of experiments repeated two or more times with an n = 60–90 worms for each condition. Kaplan-Meier log rank analysis was used to compare survival curves and to calculate the median survival. P-values <0.05 were considered to be statistically significant. **(C)** Fluorescently labelled WT, Δ*spxB* mutant and Δ*spxB;spxB*^*+*^ complemented strains of *S*. *gordonii* were exposed to L4 larvae for 2 hours and visualized by confocal microscopy. The streptococcal strains are labelled in red and are located within the intestinal lumen of the worms. Close-ups are shown in the upper right-hand corner of each image. No difference was observed in the levels of colonization in the worms between the strains. The experiment was repeated three times. **(D)** No significant difference in intestinal accumulation of WT, Δ*spxB* mutant and Δ*spxB;spxB*^*+*^ complemented strains were observed in L4 worms following a 2 hour exposure. The experiment was repeated 3 times and a total of 25 worms were disrupted for each strain of bacteria.

A previous study by Bolm, et al established that *S*. *oralis* and *S*. *mitis* caused the death of the worms via the production of H_2_O_2_ in liquid killing assays [20]. In addition to these strains, we compared the survival of worms on *S*. *gordonii*, *S*. *mutans*, and *S*. *salivarius* grown on Todd Hewitt agar supplement with yeast extract relative to non-pathogenic *E*. *coli* OP50. As observed previously, members of the mitis group rapidly killed *C*. *elegans* as opposed to *S*. *mutans*, *S*. *salivarius* and *E*. *coli* **(Fig 1A)**. The killing was abolished in the presence of 1,000 U/ml of catalase, but not on media supplemented with 100 U/ml of superoxide dismutase (**S1 and S2 Figs**). These data suggest the death of the worms is mediated by H_2_O_2_. Catalase and superoxide dismutase had no impact on the growth of the bacteria nor did they have an effect on the survival of the worms (data not shown). The production of H_2_O_2_ is mainly attributed to the pyruvate oxidase SpxB enzyme activity, which is conserved among the mitis group streptococci [35]. Therefore, we investigated the survival of the worms on *S*. *gordonii* Δ*spxB* mutant strain compared to the wild-type (WT) and complemented strain Δ*spxB;spxB*^*+*^. We observed no death of the worms on the Δ*spxB* mutant strain, whereas on the complement strain, the killing phenotype was restored as seen with the WT strain (**Fig 1B**). We did not follow the survival of the worms on the Δ*spxB* beyond 24 hours. Observing the survival of worms on Δ*spxB* mutant strain may provide insights if the mutant bacteria are completely avirulent or attenuated. In addition, continuous scoring of the WT worms on the Δ*spxB* mutant could potential identify other virulence factors of this group of bacteria. We also observed similar killing kinetics when worms were exposed to mitis group streptococci obtain from the blood of cancer patients that had undergone chemotherapy (M D. Anderson) (**S3 Fig**). Based on these data we can conclude that the mitis group streptococci mediate the killing of the worms via the production of H_2_O_2_.

### Worms are colonized with viable strains of the mitis group

We utilized two approaches to determine if the worms were colonized with streptococci. First, we visualized worms colonized with fluorescently labelled bacteria using confocal microscopy. Significant accumulation of bacteria was observed in the intestine of the worms exposed to the WT, Δ*spxB* mutant and Δ*spxB;spxB*^*+*^ complemented strains of *S*. *gordonii* (**Fig 1C**). However, this method did not confirm if the colonized bacteria were viable within the intestine of the worms. As second approach we homogenized the worms colonized with the bacteria and estimated the number of viable bacterial cells per a worm by plating. Similar levels of colonization in worms was observed for all 3 strains of *S*. *gordonii* (**Fig 1D**). These observations suggest the intestine of the worms is colonized with viable bacteria and the loss *spxB* did not cause a metabolic burden on the mutant strain as represented by the similar levels of intestinal colonization of the worms by the three strains of bacteria. More importantly, there is accumulation of the mitis group in the intestine of the worms as opposed to a previous study that showed no colonization of the worms by *S*. *pyrogenes* [22]. In this study the worms were exposed to the bacteria grown on solid media compared to *S*. *pyrogenes* grown in liquid media. This difference in growth conditions may contribute to the levels of colonization of the worms.

### Phase II response is activated in response to the pathogens

**Fig 2:**
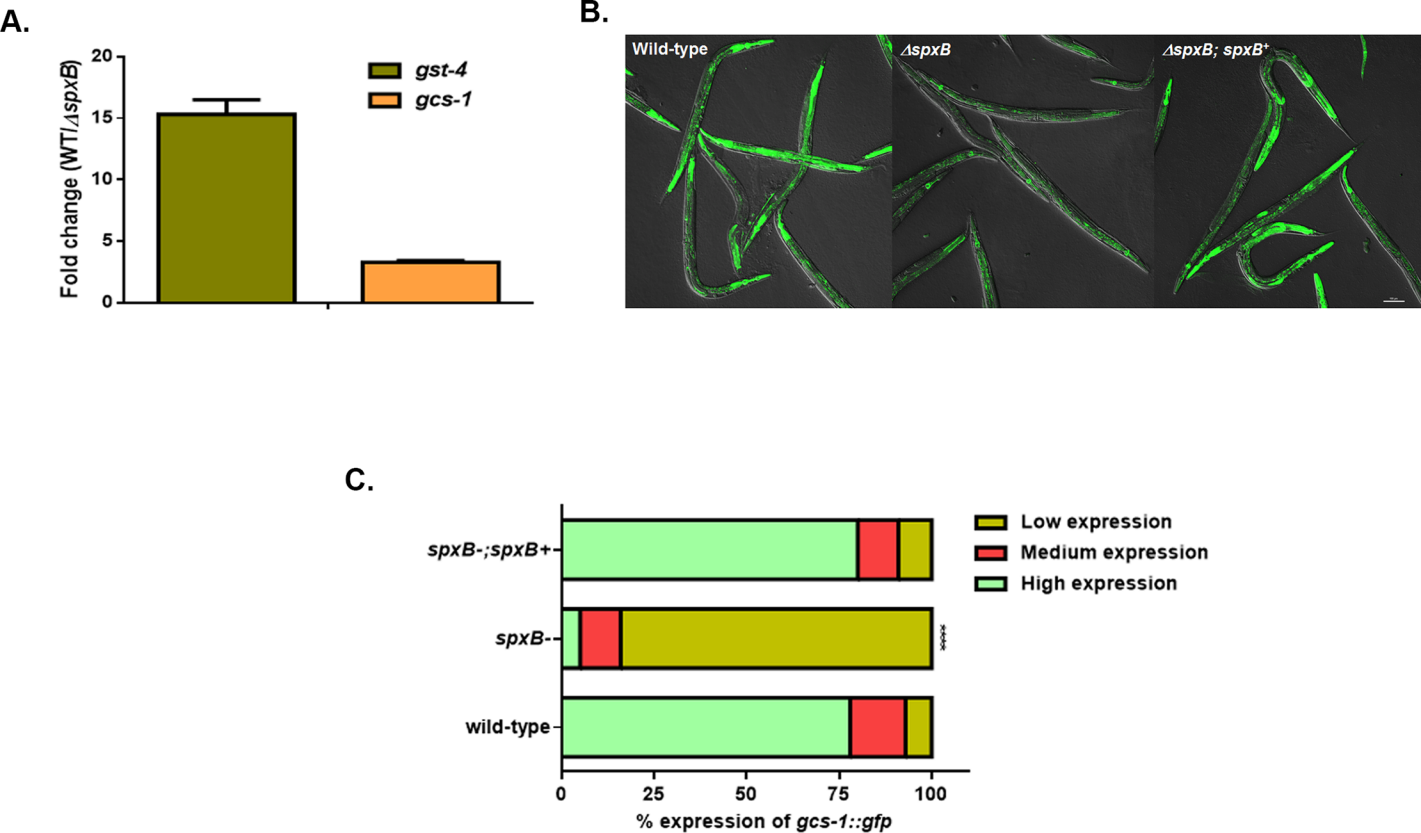
Phase II genes are upregulated in response to H_2_O_2_ produced by the mitis group streptococci. **(A)** qRT-PCR analysis of Phase II genes *gst-4* and *gcs-1* induced in worms fed for 2 hours on *S*. *gordonii* WT and Δ*spxB* mutant strains. Experiments were performed with three separate replicates; each replicate was measured in duplicate and standardized to the control gene *act-1*. Error bars represent the standard error of the mean (SEM), P<0.001. **(B)** Representative images of *gcs-1*::*gfp* expression in worms exposed to WT, Δ*spxB* mutant and Δ*spxB;spxB*^*+*^ complemented strains of *S*. *gordonii* for 2 hours. **(C)** The level of *gcs-1*::*gfp* expression and the percentage of worms in each category fed on WT, Δ*spxB* mutant and Δ*spxB;spxB*^*+*^ complemented strains of *S*. *gordonii*. A total of more than 100 worms exposed to each strain were imaged and the experiment was repeated 3 times. Significantly low levels of *gcs-1*::*gfp* expression was observed in the Δ*spxB* mutant (P<0.0001) compared to the WT and Δ*spxB;spxB*^*+*^ complement strains of *S*. *gordonii*.

In light of our findings that H_2_O_2_ mediates killing of the worms, we hypothesized that oxidative stress response genes will be upregulated against this cytotoxin. We performed RNAseq on RNA extracted from worms exposed for 2 hours to WT and Δ*spxB* mutant strains of *S*. *gordonii*. Indeed, we observed significant increase in the expression of phase II detoxification genes glutathione-S-transferases (*gst*) and γ-glutamine cysteine synthase heavy chain (*gcs-1*) in worms exposed to WT *S*. *gordonii* compared to Δ*spxB* mutant strain **(Table 1 and S1 Table)**. Expression of two important phase II genes *gst-4* and *gcs-1* was examined using quantitative real-time PCR (qRT-PCR) following exposure for 2 hours to WT and Δ*spxB* mutant strains of *S*. *gordonii*. The expression of these genes was significantly higher in the WT strain compared to the Δ*spxB* mutant strain further confirming the RNAseq data (**Fig 2A**). Furthermore, we observed similar levels of expression of these genes in L4 larvae exposed to the strains obtained from cancer patients when compared to non-pathogenic *E*. *coli* OP50 (**S4 Fig**). To further examine the expression of phase II response genes, we used a transgeneic worm strain expressing green fluorescent protein (GFP) fused to *gcs-1*. This strain was exposed to the WT, Δ*spxB* mutant and Δ*spxB;spxB*^*+*^ complement strains of *S*. *gordonii* and the levels of GFP expression was observed using a confocal microscope. Significantly higher levels of *gcs-1*::*gfp* expression was observed in the intestines of the worms exposed to the WT and complemented strains (**Fig 2B and 2C**). Whereas on the Δ*spxB* mutant, little or no expression of GFP was detected in the intestine of the worms. In addition, *gcs-1*::*gfp* levels was increased in worms fed on the strains acquired from cancer patients compared to *E*. *coli* OP50 (**S5 Fig**). Together these observations suggest the phase II genes in *C*. *elegans* are induced in response to H_2_O_2_ produced by the mitis group.

**Table 1:** Expression of phase II genes determined by RNAseq on *S*. *gordonii* WT relative to Δ*spxB* mutant strain.

Gene	Fold change in expression	p-value	FDR-corrected p-value
*gcs-1*	6.9	0	0
*gst-1*	2.1	0	0
*gst-4*	15.9	0	0
*gst-5*	4.1	4.44E-16	5.05574E-14
*gst-7*	1.7	5.99E-09	3.67613E-07
*gst-9*	3.0	0.001314	0.031406634
*gst-10*	1.7	6.46E-09	3.94E-07
*gst-12*	9.8	1.42E-06	6.33E-05
*gst-13*	4.2	0	0
*gst-19*	21.5	0.000108	0.003379
*gst-30*	19.9	3.23E-07	1.57E-05
*gst-35*	11.3	7.12E-09	4.31E-07
*gst-38*	5.3	4.34E-07	2.08E-05

### SKN-1 is required for the survival of the worms during infection

**Fig 3:**
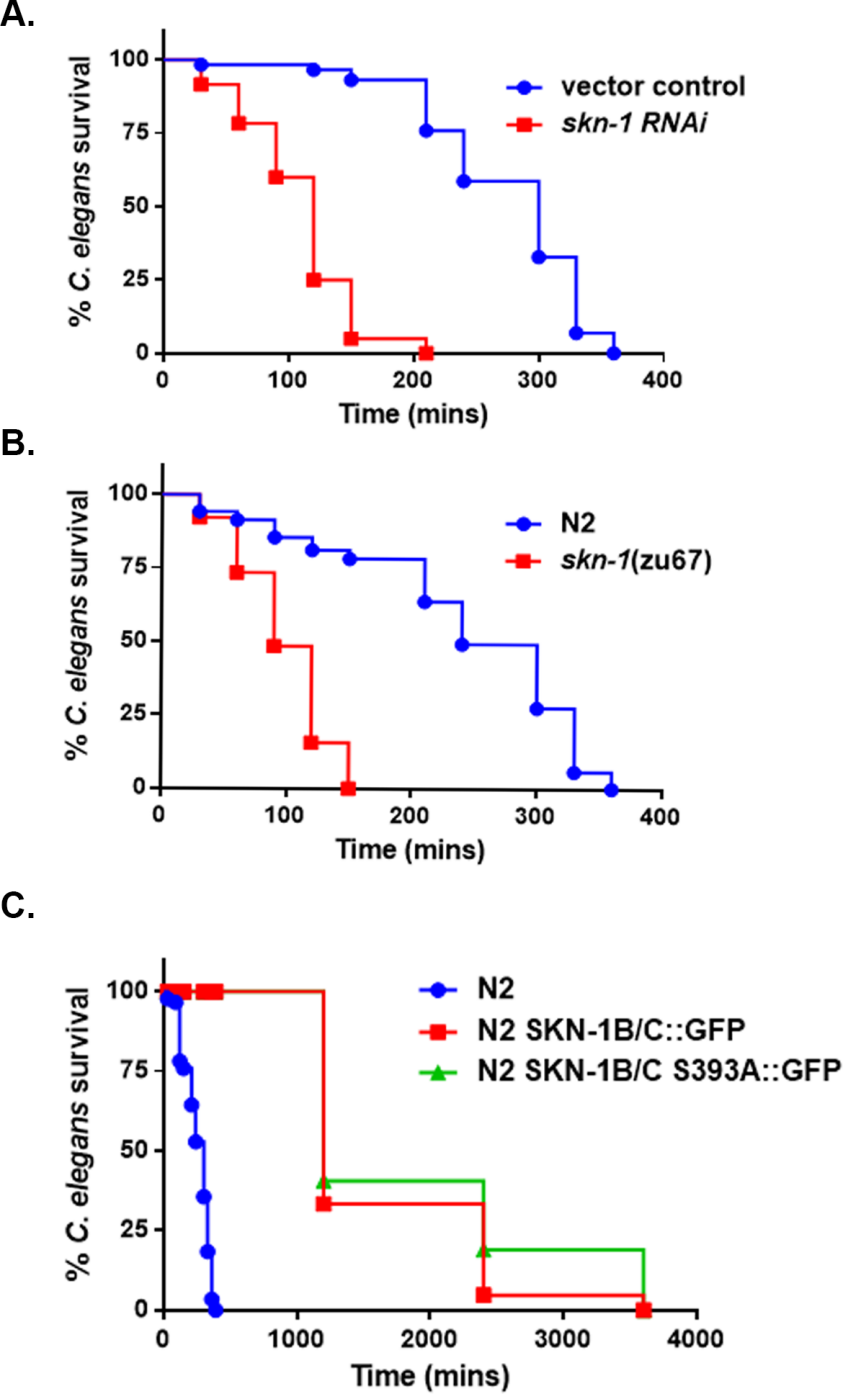
SKN-1 influences the susceptibility of the worms during infection. **(A)** Survival of vector control treated and *skn-1* knockdown worms exposed to *S*. *gordonii* on THY plates. **(B)** Survival of N2 and *skn-1*(*zu67*) mutant worms fed on THY plates containing *S*. *gordonii*. **(C)** Survival of N2 [rol-6(su1006)], N2 [SKN-1B/C::GFP[rol-6(su1006)]] and N2 [SKN-1B/C S393A::GFP[rol-6(su1006)] worms on *S*. *gordonii*. The data are representative of experiments repeated two or more times with an n = 60–90 worms for each condition. Kaplan-Meier log rank analysis was used to compare survival curves and to calculate the median survival. P-values <0.05 were considered to be statistically significant.

The oxidative stress response transcription factor SKN-1 has been shown to regulate phase II genes and is required for the survival on pathogens *Enterococcus faecalis* and *Pseudomonas aeruginosa* [32, 36]. Phase II genes *gcs-1* and *gst-4* are predominantly regulated by SKN-1 and we envisioned it is required for the survival of *C*. *elegans* on the mitis group streptococci. Knockdown of *skn-1* resulted a significant decrease in survival of the worms compared to the vector control treated worms on WT *S*. *gordonii* (**Fig 3A**). To validate this data, we compared the survival of WT worms to a *skn-1* loss of function mutant strain, *skn-1*(*zu67*). As seen in the RNAi experiment, we observed a similar killing phenotype with the *skn-1* mutant demonstrating SKN-1 influences the survival of the worms on these pathogens (**Fig 3B**). Since the loss of SKN-1 resulted in a susceptibility phenotype, we envisioned overexpression or constitutively active SKN-1 will render the worms resistant to the H_2_O_2_ produced by the mitis group. Indeed, strain SKN-1B/C::GFP carrying extra copies of *skn-1* and strain SKN-1B/C S393A::GFP expressing a constitutively active form of SKN-1 [32, 37] exhibited increased resistance compared to the N2 worms in the presence of the WT *S*. *gordonii* (**Fig 3C**). There was no significant difference between the survival of the SKN-1B/C::GFP and the SKN-1B/C S393A::GFP strains on the pathogen. Taken together, these data show loss of *skn-1* is detrimental to the survival of the worms, whereas increase of SKN-1 activity provides a protective function against these pathogens.

### Activation of SKN-1 requires the p38 MAPK pathway in response to H_2_O_2_ produced by the mitis group streptococci

**Fig 4:**
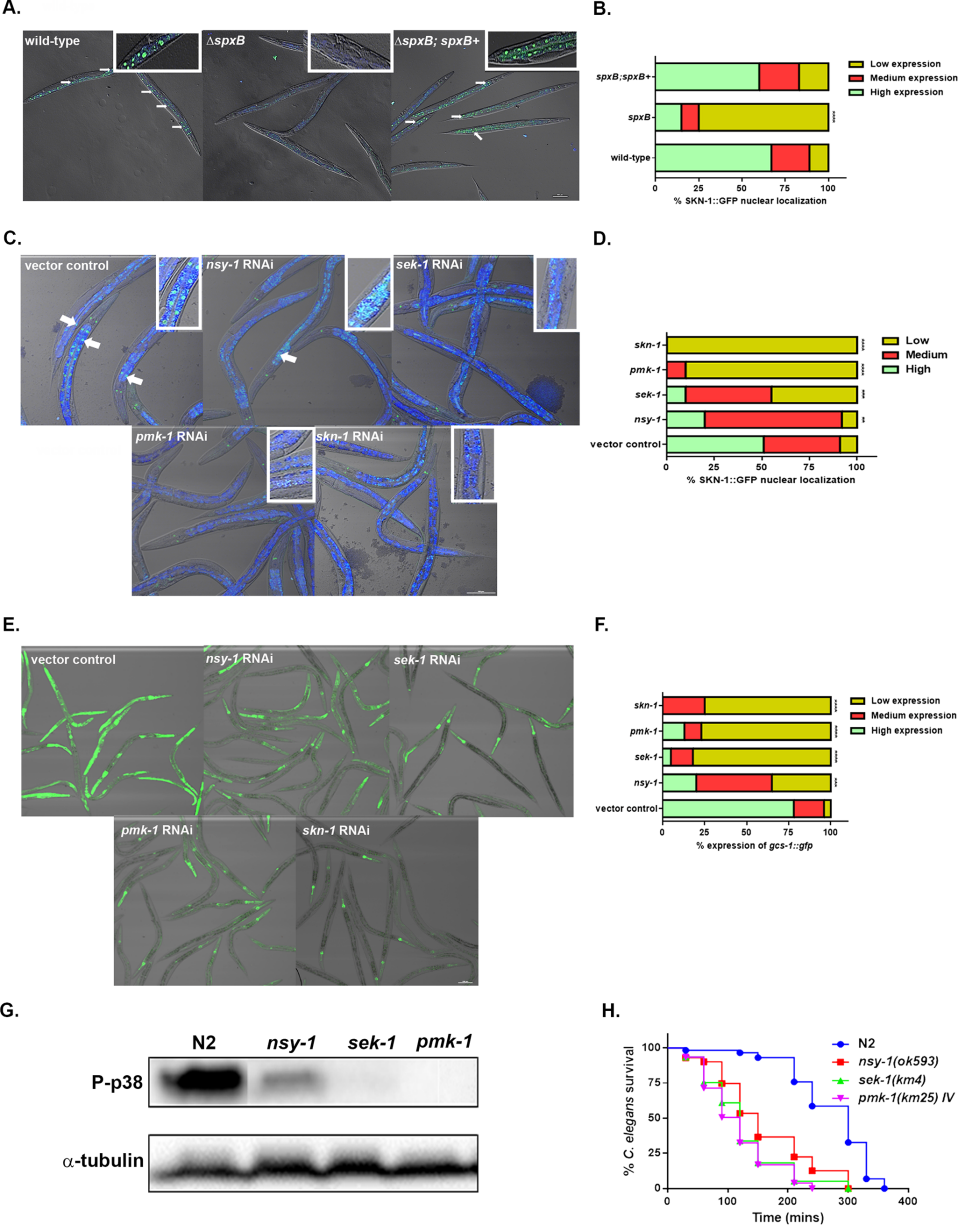
SKN-1 activation is dependent on the p38 MAPK pathway in response to the pathogens. **(A)** Representative images of the localization of SKN-1B/C::GFP in worms exposed to the WT, Δ*spxB* mutant and Δ*spxB;spxB*^*+*^ complement strains of *S*. *gordonii*. Close-ups are shown in the upper right-hand corner of each image. (**B)** The degree of nuclear localization of SKN-1B/C::GFP and the percentage of worms in each category fed on WT, Δ*spxB* mutant and Δ*spxB;spxB*^*+*^ complement strains of *S*. *gordonii*. A total of more than 100 worms exposed to each strain were imaged and the experiment was repeated 3 times. Significantly lower levels of nuclear localization of SKN-1B/C::GFP was observed in the Δ*spxB* mutant (P<0.0001) compared to the WT and Δ*spxB;spxB*^*+*^ complement strains of *S*. *gordonii*. **(C)** Representative images of the localization of SKN-1B/C::GFP in *nsy-1*, *sek-1*, *pmk-1*, *skn-1* knockdown and vector control treated worms on *S*. *gordonii*. Close-ups are shown in the upper right-hand corner of each image. **(D)** The degree of SKN-1B/C::GFP nuclear localization and the percentage of worms in each category fed on *nsy-1*, *sek-1*, *pmk-1*, *skn-1* knockdown and vector control treated worms on *S*. *gordonii*. A total of more than 100 worms exposed to each strain were imaged and the experiment was repeated 3 times. Significantly lower levels of nuclear localization of SKN-1B/C::GFP was observed in the *nsy-1*(P<0.01), *sek-1* (P<0.001), *pmk-1* (P<0.0001), *skn-1* knockdown (P<0.0001) compared to the vector control treated worms on *S*. *gordonii*. **(E)** Representative images of *gcs-1*::*gfp* expression in *nsy-1*, *sek-1*, *pmk-1*, *skn-1* knockdown and vector control treated worms on *S*. *gordonii*. **(F)** The level of *gcs-1*::*gfp* expression and the percentage of worms in each category fed on *nsy-1*, *sek-1*, *pmk-1*, *skn-1* knockdown and vector control treated worms on *S*. *gordonii*. A total of more than 100 worms exposed to each strain was imaged and the experiment was repeated 3 times. Significantly lower levels of *gcs-1*::*gfp* expression was observed in the *nsy-1*(P<0.001), *sek-1* (P<0.0001), *pmk-1* (P<0.0001), *skn-1* knockdown (P<0.0001) compared to the vector control treated worms on *S*. *gordonii*. **(G)** Western blot was used to analyze the level of phosphorylation of PMK-1 in N2, *nsy-1*, *sek-1* and *pmk-1* mutant worms exposed to *S*. *gordonii* for 2 hours. Phosphorylation of PMK-1 was partial reduced in *nsy-1* mutant worms, while in the *sek-1* mutant worms phospho-p38 was completely absent. *pmk-1* mutant worms was used as a negative control and α-tubulin as a loading control. **(H)** Survival of N2 and *nsy-1*(*ok593)*, *sek-1*(*km4)* and *pmk-1*(*km25*) IV L4 larvae when fed on THY plates containing *S*. *gordonii*. The data are representative of experiments repeated two or more times with an n = 60–90 worms for each condition. Kaplan-Meier log rank analysis was used to compare survival curves and to calculate the median survival. P-values <0.05 were considered to be statistically significant.

Previous work has established SKN-1 activity is increased in the presence of *E*. *faecalis* and *P*. *aeruginosa* [32, 36]. The activity of SKN-1 can be determined by detecting the localization of this transcription factor into the intestinal nuclei using a transgenic line expressing SKN-1 fused to GFP. Using confocal microscopy, we observed greater levels of SKN-1B/C::GFP localization in the intestinal nuclei in worms on the WT and complementary strains of *S*. *gordonii* (**Fig 4A and 4B**). Significantly lower levels of SKN-1 localization was detected in worms exposed to the Δ*spxB* mutant. High levels of localization of SKN-1 was also observed when SKN-1B/C::GFP expressing worms were exposed to the clincial isolates (**S6 Fig**). These data further confirms SKN-1 is activated by H_2_O_2_ produced by this group of organisms. The activation of SKN-1 has been shown to regulated by p38 MAPK pathway in response to oxidative stresses and to pathogens. Using the SKN-1B/C::GFP transgenic worms, we knockdown components of the p38 MAPK pathway mainly *nsy-1*, *sek-1*, *pmk-1* and *skn-1* and determined the localization of SKN-1 on WT *S*. *gordonii* (**Fig 4C and 4D**). SKN-1 localization was significantly reduced in the *sek-1*, *pmk-1* and *skn-1* knockdown worms compared to the vector control treated worms. Partial reduction of SKN-1 localization was observed in *nsy-1* knockdown on *S*. *gordonii* suggesting an additional pathway activates SKN-1 via the interaction with SEK-1. In addition, we determine the expression of *gcs-1*::*gfp* in *nsy-1*, *sek-1*, *pmk-1* and *skn-1* knockdown worms relative to the vector control treated worms *(***Fig 4E and 4F**). Significant reduction in expression of *gcs-1*::*gfp* was observed in the *sek-1*, *pmk-1* and *skn-1* knockdown worms compared to the vector control. However, the *gcs-1*::*gfp* expression in *nsy-1* knockdown worms was not comparable to the *sek-1*, *pmk-1* and *skn-1* knockdown worms, but significantly reduced relative to the vector control treated worms. To further determine if the p38 MAPK pathway is activated in response to H_2_O_2_ produced by the mitis group, we determined the level of phosphorylation of PMK-1 in *nsy-1*, *sek-1*, *and pmk-1* mutant worms compared to N2 worms by western blot (**Fig 4G**). We observed no phosphorylation of PMK-1 in the *sek-1* worms, whereas the level of phosphorylation in the *nsy-1* mutant worms was significantly lower compared to the N2 worms on *S*. *gordonii*. *pmk-1* mutant worms were used as a negative control. This data further indicates the p38 MAPK pathway is activated in response to the mitis group streptococci. However, the phosphorylation of PMK-1 is partially dependent on NSY-1 and further supports the SKN-1 localization data. A recent study showed the mitochondrial chaperone HSP-60 physically binds and stabilizes SEK-1, thereby activating PMK-1 and increasing immunity in response to *P*. *aeruginosa* [38]. Perhaps, HSP-60 interacts with SEK-1 to activate SKN-1 via PMK-1 in response to H_2_O_2_ produced by the mitis group streptococci. Future studies will be needed to address the activation of SKN-1 by HSP-60 via the p38 MAPK pathway.

We next determined if the loss of *nsy-1*, *sek-1* and *pmk-1* reduced the survival of the worms relative to the WT strain when exposed to *S*. *gordonii*. A significant decrease in the survival of the *nsy-1*, *sek-1* and *pmk-1* mutant strains was observed compared to the N2 strain. The data shows the p38 MAPK pathway is required for the survival of *C*. *elegans* in the presence of *S*. *gordonii*.

### IRE-1-TRAF (*trf-1*) is not required for the activation of SKN-1 in response to the mitis group

**Fig 5:**
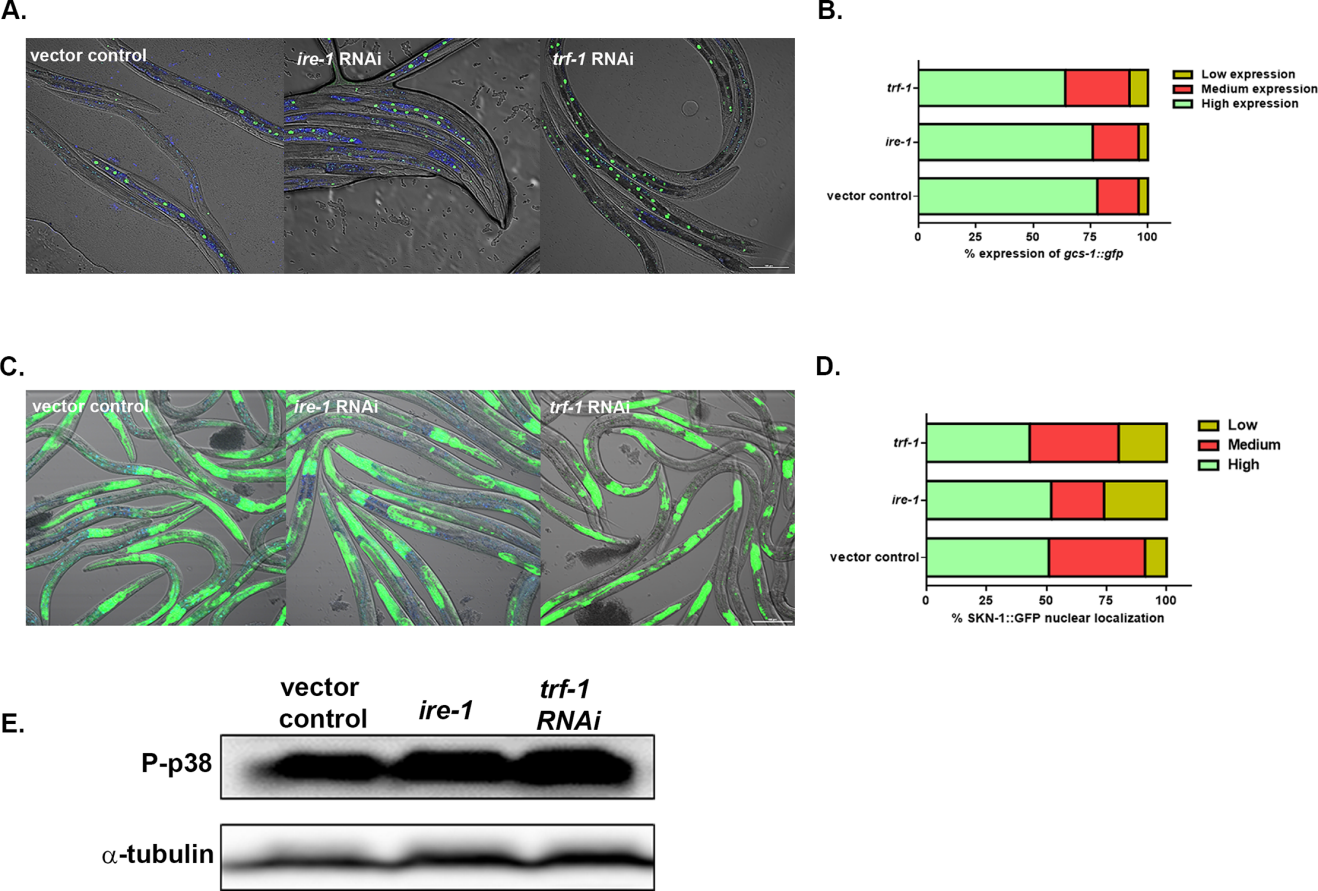
Activation of SKN-1 does not require IRE-1-TRAF (*trf-1*), when exposed to the *S*. *gordonii*. **(A)** Representative images of the localization of SKN-1B/C::GFP in *ire-1*, *trf-1* knockdown and vector control treated worms on *S*. *gordonii* for 2 hours. **(B)** The degree of SKN-1B/C::GFP nuclear localization and the percentage of worms in each category fed on *ire-1*, *trf-1* knockdown and vector control treated worms on *S*. *gordonii*. A total of more than 100 worms exposed to each strain were imaged and the experiment was repeated 3 times. Similar levels of nuclear localization of SKN-1B/C::GFP was observed in the *ire-1*, *trf-1* knockdown and vector control treated worms on *S*. *gordonii*. **(C)** Representative images of *gcs-1*::*gfp* expression in *nsy-1*, *sek-1*, *pmk-1*, *skn-1* knockdown and vector control treated worms on *S*. *gordonii*. **(D)** The level of *gcs-1*::*gfp* expression and the percentage of worms in each category fed on *ire-1*, *trf-1* knockdown and vector control treated worms on *S*. *gordonii* for 2 hours. A total of more than 100 worms exposed to each strain were imaged and the experiment was repeated 3 times. No difference of *gcs-1*::*gfp* expression was observed in the *ire-1*, *trf-1* knockdown worms compared to the vector control treated worms on *S*. *gordonii*. **(E)** Western blot was used to analyze the level of phosphorylation of PMK-1 in vector control treated worms, *ire-1* mutant worms and *trf-1* knockdown worms exposed to *S*. *gordonii* for 2 hours. No significant differences was observed in the level of phosphorylation of PMK-1 in the *ire-1* mutant worms and *trf-1* knockdown worms compared to the vector control treated worms. α-tubulin was used as a loading control. The experiment was performed three times.

A recent study uncovered an unexpected function for the ER transmembrane protein IRE-1 in redox-regulated cytoplasmic signaling. On treatment with arsenite, IRE-1 and NSY-1 form a complex via the participation of TRAF-2 (*trf-1*) resulting in the activation of the p38 MAPK pathway [33]. In light of this discovery we investigated if IRE-1 and TRAF-2 (*trf-1*) are required for the activation of the p38 signaling in response to *S*. *gordonii*. We first determined the localization of SKN-1B/C::GFP in *ire-1* and *trf-1* knockdown worms relative to the vector treated worms on the pathogen (**Fig 5A and 5B**). The percentage of SKN-1 localization was similar in *ire-1* and *trf-1* knockdown worms compared to the vector control treated worms. We next determined the expression of *gcs-1*::*gfp* in *ire-1* and *trf-1* knockdown worms compared to the vector control treated worms on *S*. *gordonii* (**Fig 5C and 5D**). No significant difference in expression of *gcs-1*::*gfp* was observed between the knockdowns and the vector control treated worms. We further investigated if the levels of phosphorylation of PMK-1 is influenced by IRE-1 and TRAF-2 in the presence of *S*. *gordonii (***Fig 5E**). No difference in the level of phosphorylation of PMK-1 by western blot was observed in the *ire-1* mutant strain and the *trf-1* knockdown strain compared to the vector control treated worms.

**Fig 6:**
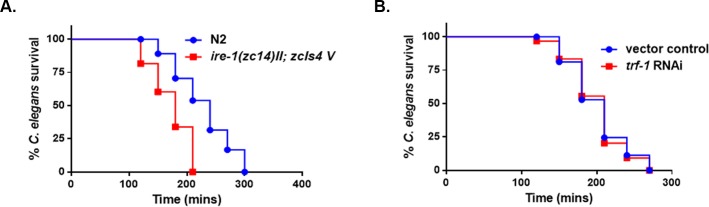
IRE-1 is required for the survival of the worms on *S*. *gordonii*. **(A)** Survival of N2 and *ire-1(zc14) II; zcIs4 V* L4 larvae when fed on THY plates containing *S*. *gordonii*. **(B)** Survival of vector control treated and *trf-1* knockdown worms exposed to *S*. *gordonii* on THY plates. The data are representative of experiments repeated two or more times with an n = 60–90 worms for each condition. Kaplan-Meier log rank analysis was used to compare survival curves and to calculate the median survival. P-values <0.05 were considered to be statistically significant.

Lastly, we determined the survival of the *ire-1* mutant strain relative to the N2—strain and the *trf-1* knockdown strain relative to the vector control treated worms on the pathogen (**Fig 6A and 6B**). A significant decrease in survival was observed in the *ire-1* mutant strain compared to WT strain. However, no difference in survival was seen between the *trf-1* knockdown and vector control treated worms in response to *S*. *gordonii*. Taken together the data suggests the phosphorylation of PMK-1 does not require the IRE-1 TRAF-2 complex, but is activated by a parallel pathway. Moreover, IRE-1 may be required for induction of the ER stress response when exposed to *S*. *gordonii*. Numerous studies have shown that oxidative stress induces ER stress and the pattern of UPR activation is dependent on the cell type [39–41]. The Unfolded protein response (UPR) may indeed play an important role in the protection of the worms in response to oxidative stress caused by the mitis group. Further investigation is required to elucidate the parallel pathway for p38 phosphorylation and the role of the UPR in response to the streptococcal H_2_O_2_.

### The activation of the p38 MAPK pathway in response to *S*. *gordonii* is not dependent on Ce-DUOX-1/BLI-3

**Fig 7:**
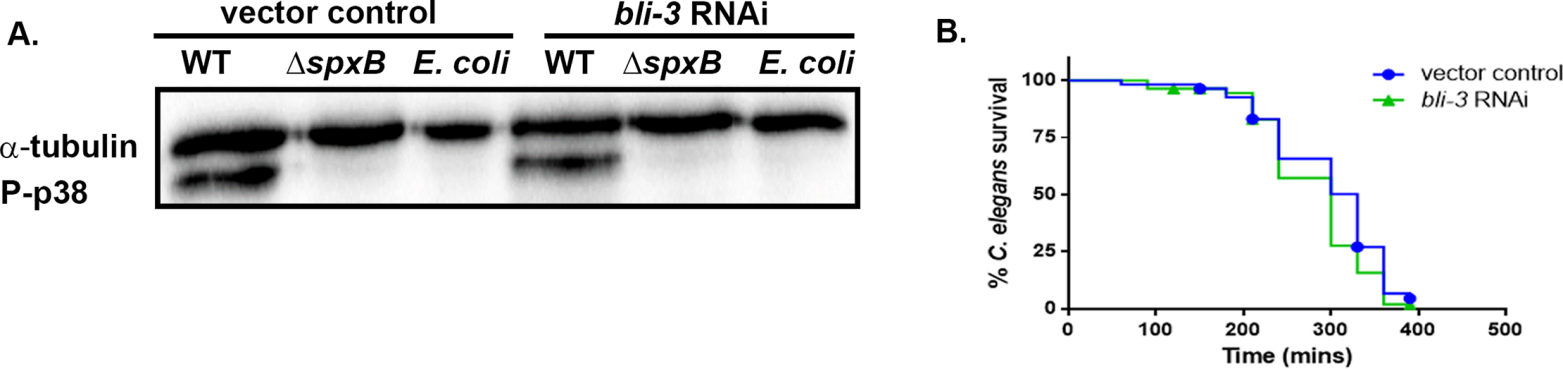
Activation of the p38 MAPK pathway is not dependent on Ce-DUOX-1/BLI-3. **(A)** Western blot was used to analyze the level of phosphorylation of PMK-1 in *bli-3* knockdown and vector control treated worms exposed to *S*. *gordonii* WT, Δ*spxB* and *E*. *coli* OP50 for 2 hours. No significant difference in phospho-p38 levels was observed in *bli-3* knockdown worms relative to vector control treated worms. Phosphorylation of PMK-1 was absent in vector control treated and *bli-3* knockdowns worms on the *S*. *gordonii* Δ*spxB* mutant strain and *E*. *coli* OP50. α-tubulin was used as a loading control. The experiment was repeated 3 times **(B)** Survival of vector control treated and *bli-3* knockdown worms exposed to *S*. *gordonii* on THY plates. The data are representative of experiments repeated two or more times with an n = 60–90 worms for each condition. Kaplan-Meier log rank analysis was used to compare survival curves and to calculate the median survival. P-values <0.05 were considered to be statistically significant.

The activation of SKN-1 in response to pathogens and arsenite has shown to be mediated by ROS released by the dual oxidase Ce-Duox1/BLI-3 [32, 33]. We examined if indeed H_2_O_2_ produced by Ce-Duox1/BLI-3 in response to *S*. *gordonii* contributes to the activation of the p38 MAPK pathway. To test this, we determine the levels of phosphorylation of PMK-1 by western blot in *bli-3* knockdown and vector control treated worms exposed to *S*. *gordonii* WT, Δ*spxB* and *E*. *coli* OP50. No significant change in the level of phospho-p38 was observed between the *bli-3* knockdown and vector control treated worms on WT *S*. *gordonii* (**Fig 7A**). Furthermore, we detected significantly lower levels of phospho-p38 in *bli-3* knockdown and vector control treated worms fed on the Δ*spx*B mutant strain of *S*. *gordonii* or *E*. *coli* OP50. The data suggests the activation of the p38 MAPK pathway in response to *S*. *gordonii* is not dependent on Ce-Duox1/BLI-3. Furthermore, to confirm if Ce-Duox1/BLI-3 is required for the survival of the worms on *S*. *gordonii*, we determine the susceptibility of *bli-3* knockdown and vector control treated L4 larvae. There was no significant difference in survival between *bli-3* knockdown and the vector control treated worms on the pathogen (**Fig 7B**). Taken together, these findings suggest Ce-Duox1/BLI-3 is not activated to produce ROS in response to *S*. *gordonii* and hence does not contribute to the induction of p38 MAPK pathway.

## Conclusion

In this study, we demonstrate the oxidative stress response transcription factor SKN-1 is required for the survival of the worms when exposed to the mitis group streptococci. Furthermore, we demonstrate H_2_O_2_ produced by this group of bacteria activates SKN-1 via the p38 MAPK pathway. However, the activation of p38 signaling is not via the IRE-1-NSY-1 complex and is not dependent on Ce-Duox1/BLI-3, suggesting that a parallel mechanism senses H_2_O_2_ produced by the mitis group and activates p38 via NSY-1. In addition, our data suggests an NSY-1 independent mechanism activates the p38 pathway through SEK-1. Even though IRE-1 does not initiate the p38 signaling, our data indicates this kinase is required for the survival of the worms in the presence of the mitis group. Defining these mechanisms will be important areas of future research. Using *C*. *elegans* as a model host, we are able to determine the significance of H_2_O_2_ produced by the mitis group of streptococci on cellular mechanisms in context of a whole organism.

## Supporting information

S1 Table List of *C*. *elegans* strains used in this study. (DOCX)

S2 Table Fold changes of genes from RNA Seq analysis. (XLSX)

S1 Fig Kiiling of the worms by the mitis group streptococci is abolished in the presence of catalase. Survival of N2 L4 larvae exposed to *S*. *gordonii*, *S*. *oralis*, *S*. *mitis*, *S*. *salivarius S*. *mutans* and *E*. *coli* OP50 on THY plates in the presence of 1000U of catalase. The data are representative of experiments repeated two or more times with an n = 60–90 worms for each condition. Kaplan-Meier log rank analysis was used to compare survival curves and to calculate the median survival. P-values <0.05 were considered to be statistically significant.
(PDF)

S2 Fig Killing of the worms by the mitis group streptococci is not influenced by the presence of superoxide dismutase. Survival of N2 L4 larvae exposed to *S*. *gordonii*, *S*. *oralis*, *S*. *mitis*, *S*. *salivarius*, *S*. *mutans* and *E*. *coli* OP50 on THY plates supplemented with 100U of superoxide dismutase. The data are representative of experiments repeated two or more times with an n = 60–90 worms for each condition. Kaplan-Meier log rank analysis was used to compare survival curves and to calculate the median survival. P-values <0.05 were considered to be statistically significant.
(PDF)

S3 Fig Clinical isolates of the mitis group streptococci rapidly kill the worms. Survival of N2 L4 larvae exposed to *S*. *oralis* (VGS#3), *S*. *oralis* (VGS#4), *S*. *mitis* (VGS#10), *S*. *mitis* (VGS#13) and *E*. *coli* OP50 on THY plates. The data are representative of experiments repeated two or more times with an n = 60–90 worms for each condition. Kaplan-Meier log rank analysis was used to compare survival curves and to calculate the median survival. P-values <0.05 were considered to be statistically significant.
(PDF)

S4 Fig Phase II genes are upregulated in response to H_2_O_2_ produced by clinical isolates of the mitis group streptococci. qRT-PCR analysis of Phase II genes *gst-4* and *gcs-1* induced in worms fed for 2 hours on *S*. *oralis* (VGS#3), *S*. *mitis* (VGS#10) and *E*. *coli* OP50. Experiments were performed with three separate replicates; each replicate was measured in duplicate and standardized to the control gene *act-1*. Error bars represent the standard error of the mean (SEM), P<0.001.
(PDF)

S5 Fig Significantly high levels of *gcs-1*::*gfp* expression in worms exposed to the clinical isolates of the mitis group streptococci. Representative images of *gcs-1*::*gfp* expression in worms exposed to *S*. *oralis* (VGS#3), *S*. *mitis* (VGS#10) and *E*. *coli* OP50.for 2 hours. The level of *gcs-1*::*gfp* expression and the percentage of worms in each category fed on *S*. *oralis* (VGS#3), *S*. *mitis* (VGS#10) and *E*. *coli* OP50. A total of more than 100 worms exposed to each strain were imaged and the experiment was repeated 3 times. Significantly high levels of *gcs-1*::*gfp* expression in worms was observed on *S*. *oralis* (VGS#3) and *S*. *mitis* (VGS#10) (P<0.0001) compared to *E*. *coli* OP50.
(PDF)

S6 Fig Significantly high levels of SKN-1B/C::GFP localization in worms exposed to the clinical isolates of the mitis group streptococci. Representative images of the localization of SKN-1B/C::GFP in worms exposed to t *S*. *oralis* (VGS#3), *S*. *mitis* (VGS#10) and *E*. *coli* OP50.for 2 hours. The degree of nuclear localization of SKN-1B/C::GFP and the percentage of worms in each category fed on *S*. *oralis* (VGS#3), *S*. *mitis* (VGS#10) and *E*. *coli* OP50. A total of more than 100 worms exposed to each strain were imaged and the experiment was repeated 3 times. Significantly high levels of nuclear localization of SKN-1B/C::GFP were observed in worms on *S*. *oralis* (VGS#3) and *S*. *mitis* (VGS#10) strains (P<0.0001) compared to *E*. *coli* OP50.
(PDF)
