# Supplementary material for: The activation of the oxidative stress response transcription factor SKN-1 in Caenorhabditis elegans by mitis group streptococci
Source: PLoS One. 2018 Aug 16;13(8):e0202233. doi: 10.1371/journal.pone.0202233 (PMC6095534; doi:10.1371/journal.pone.0202233)
Supplement: S1 Table — (DOCX) [file pone.0202233.s001.docx]

**S1 Table. List of *C. elegans* strains used in this study**

| **Strain** | **Genotype** | **Transgene** | **Source** |
| --- | --- | --- | --- |
| N2 | *C. elegans* wild isolate |  | CGC |
| EU1 | *skn-1*(zu67) IV/nT1 [unc-?(n754) let-?] (IV;V) |  | CGC |
| VC390 | *nsy-1*(ok593) II |  | CGC |
| KU4 | *sek-1*(km4) X |  | CGC |
| KU25 | *pmk-1*(km25) IV |  | CGC |
| SJ30 | *ire-1*(zc14) II; zcIs4 V |  | CGC |
| LD1171 | ldIs3 | gcs-1p::GFP + rol-6(su1006) | CGC |
| LD002 | IdIs1 | SKN-1B/C::GFP + rol-6(su1006) | Keith Blackwell |
| LD1252 | N2 | SKN -1B/C S393A::GFP + rol-6(su1006) | Keith Blackwell |
| GF15 | N2 | rol-6(su1006) | Danielle Garsin |
