# Supplementary material for: The activation of the oxidative stress response transcription factor SKN-1 in Caenorhabditis elegans by mitis group streptococci
Source: PLoS One. 2018 Aug 16;13(8):e0202233. doi: 10.1371/journal.pone.0202233 (PMC6095534; doi:10.1371/journal.pone.0202233)
Supplement: S1 Fig — Survival of N2 L4 larvae exposed to S. gordonii, S. oralis, S. mitis, S. salivarius S. mutans and E. coli OP50 on THY plates in the presence of 1000U of catalase. The data are representative of experiments repeated two or more times with an n = 60–90 worms for each condition. Kaplan-Meier log rank analysis was used to compare survival curves and to calculate the median survival. P-values <0.05 were considered to be statistically significant. (PDF) [file pone.0202233.s003.pdf]

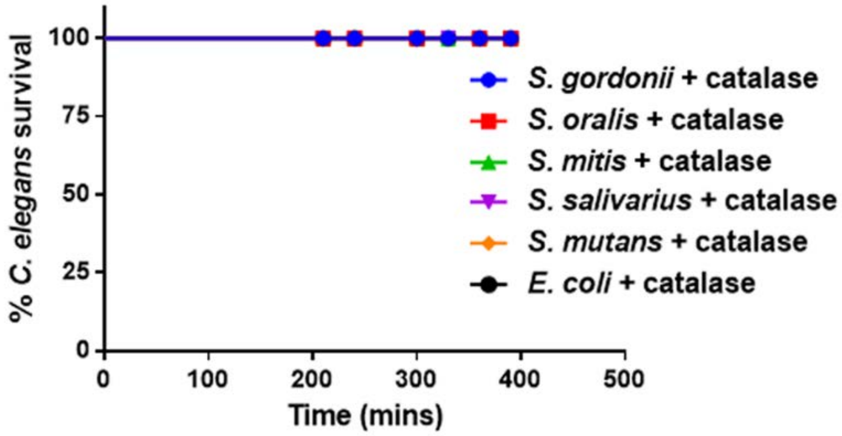

S1 Fig. Kiiling of the worms by the mitis group streptococci is abolished in the presence of catalase. Survival of N2 L4 larvae exposed to *S. gordonii*, *S. oralis*, *S. mitis*, *S. salivarius S. mutans* and *E. coli* OP50 on THY plates in the presence of 1000U of catalase. The data are representative of experiments repeated two or more times with an n = 60 – 90 worms for each condition. Kaplan-Meier log rank analysis was used to compare survival curves and to calculate the median survival. P-values <0.05 were considered to be statistically significant.
