# Supplementary material for: The activation of the oxidative stress response transcription factor SKN-1 in Caenorhabditis elegans by mitis group streptococci
Source: PLoS One. 2018 Aug 16;13(8):e0202233. doi: 10.1371/journal.pone.0202233 (PMC6095534; doi:10.1371/journal.pone.0202233)
Supplement: S3 Fig — Survival of N2 L4 larvae exposed to S. oralis (VGS#3), S. oralis (VGS#4), S. mitis (VGS#10), S. mitis (VGS#13) and E. coli OP50 on THY plates. The data are representative of experiments repeated two or more times with an n = 60–90 worms for each condition. Kaplan-Meier log rank analysis was used to compare survival curves and to calculate the median survival. P-values <0.05 were considered to be statistically significant. (PDF) [file pone.0202233.s005.pdf]

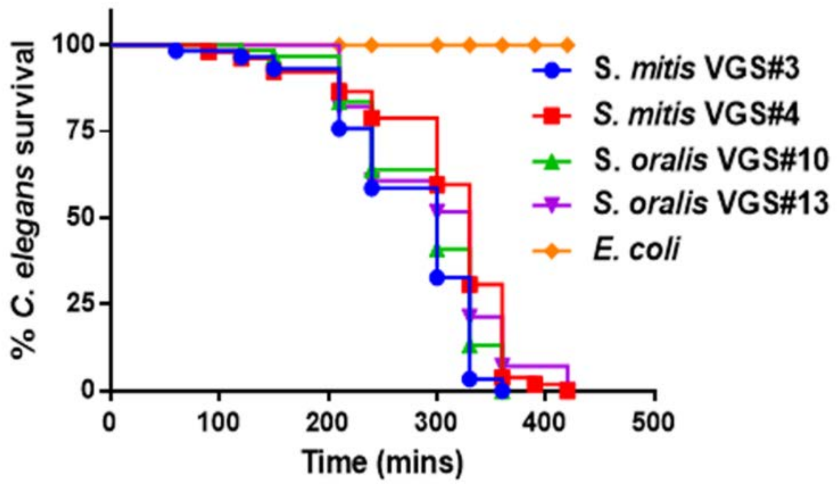

S3 Fig. Clinical isolates of the mitis group streptococci rapidly kill the worms. Survival of N2 L4 larvae exposed to *S. oralis* (VGS#3), *S. oralis* (VGS#4), *S. mitis* (VGS#10), *S. mitis* (VGS#13) and *E. coli* OP50 on THY plates. The data are representative of experiments repeated two or more times with an n = 60 – 90 worms for each condition. Kaplan-Meier log rank analysis was used to compare survival curves and to calculate the median survival. P-values <0.05 were considered to be statistically significant.
