# Supplementary material for: The activation of the oxidative stress response transcription factor SKN-1 in Caenorhabditis elegans by mitis group streptococci
Source: PLoS One. 2018 Aug 16;13(8):e0202233. doi: 10.1371/journal.pone.0202233 (PMC6095534; doi:10.1371/journal.pone.0202233)
Supplement: S4 Fig — qRT-PCR analysis of Phase II genes gst-4 and gcs-1 induced in worms fed for 2 hours on S. oralis (VGS#3), S. mitis (VGS#10) and E. coli OP50. Experiments were performed with three separate replicates; each replicate was measured in duplicate and standardized to the control gene act-1. Error bars represent the standard error of the mean (SEM), P<0.001. (PDF) [file pone.0202233.s006.pdf]

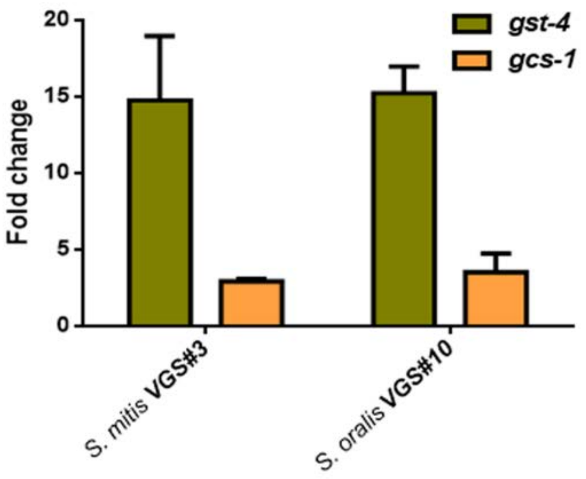

S4 Fig. Phase II genes are upregulated in response to H_2O_2 produced by clinical isolates of the mitis group streptococci. qRT-PCR analysis of Phase II genes *gst-4* and *gcs-1* induced in worms fed for 2 hours on *S. oralis* (VGS#3), *S. mitis* (VGS#10) and *E. coli* OP50. Experiments were performed with three separate replicates; each replicate was measured in duplicate and standardized to the control gene *act-1*. Error bars represent the standard error of the mean (SEM), $P<0.001$.
