# Supplementary material for: The activation of the oxidative stress response transcription factor SKN-1 in Caenorhabditis elegans by mitis group streptococci
Source: PLoS One. 2018 Aug 16;13(8):e0202233. doi: 10.1371/journal.pone.0202233 (PMC6095534; doi:10.1371/journal.pone.0202233)
Supplement: S5 Fig — Representative images of gcs-1::gfp expression in worms exposed to S. oralis (VGS#3), S. mitis (VGS#10) and E. coli OP50.for 2 hours. The level of gcs-1::gfp expression and the percentage of worms in each category fed on S. oralis (VGS#3), S. mitis (VGS#10) and E. coli OP50. A total of more than 100 worms exposed to each strain were imaged and the experiment was repeated 3 times. Significantly high levels of gcs-1::gfp expression in worms was observed on S. oralis (VGS#3) and S. mitis (VGS#10) (P<0.0001) compared to E. coli OP50. (PDF) [file pone.0202233.s007.pdf]

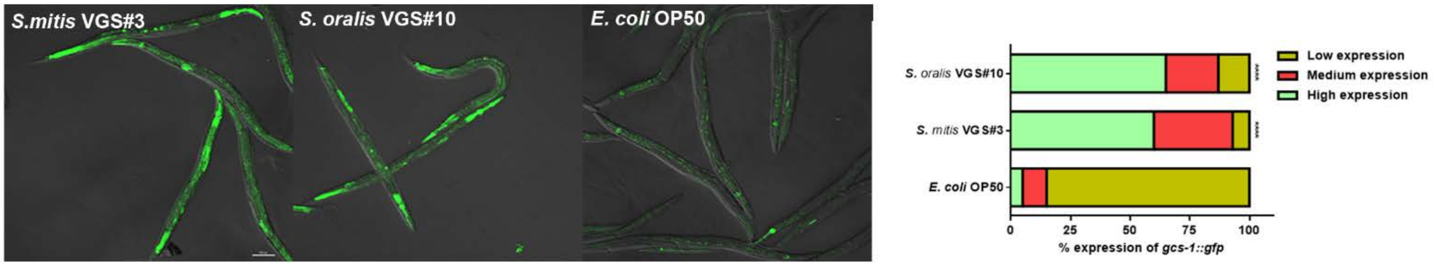

S5 Fig. Significantly high levels of *gcs-1*::*gfp* expression in worms exposed to the clinical isolates of the mitis group streptococci. Representative images of *gcs-1*::*gfp* expression in worms exposed to *S. oralis* (VGS#3), *S. mitis* (VGS#10) and *E. coli* OP50.for 2 hours. The level of *gcs-1::gfp* expression and the percentage of worms in each category fed on *S. oralis* (VGS#3), *S. mitis* (VGS#10) and *E. coli* OP50. A total of more than 100 worms exposed to each strain were imaged and the experiment was repeated 3 times. Significantly high levels of *gcs-1::gfp* expression in worms was observed on *S. oralis* (VGS#3) and *S. mitis* (VGS#10) (P<0.0001) compared to *E. coli* OP50.
