# Supplementary material for: The activation of the oxidative stress response transcription factor SKN-1 in Caenorhabditis elegans by mitis group streptococci
Source: PLoS One. 2018 Aug 16;13(8):e0202233. doi: 10.1371/journal.pone.0202233 (PMC6095534; doi:10.1371/journal.pone.0202233)
Supplement: S6 Fig — Representative images of the localization of SKN-1B/C::GFP in worms exposed to t S. oralis (VGS#3), S. mitis (VGS#10) and E. coli OP50.for 2 hours. The degree of nuclear localization of SKN-1B/C::GFP and the percentage of worms in each category fed on S. oralis (VGS#3), S. mitis (VGS#10) and E. coli OP50. A total of more than 100 worms exposed to each strain were imaged and the experiment was repeated 3 times. Significantly high levels of nuclear localization of SKN-1B/C::GFP were observed in worms on S. oralis (VGS#3) and S. mitis (VGS#10) strains (P<0.0001) compared to E. coli OP50. (PDF) [file pone.0202233.s008.pdf]

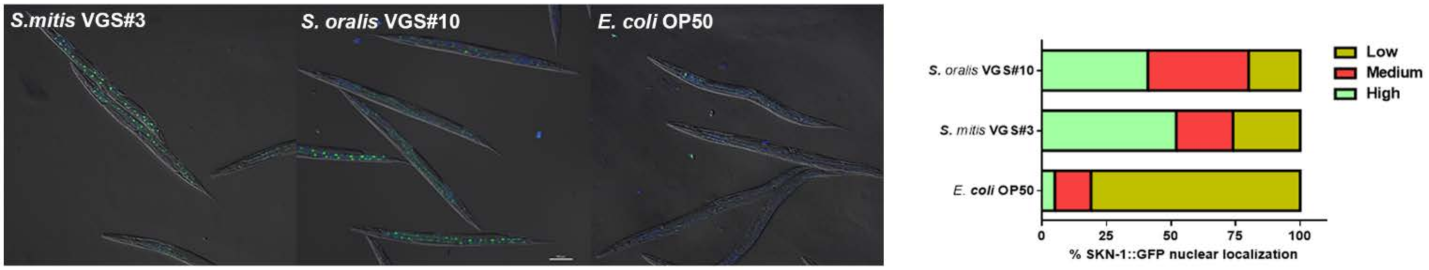

S6 Fig. Significantly high levels of SKN-1B/C::GFP localization in worms exposed to the clinical isolates of the mitis group streptococci. Representative images of the localization of SKN-1B/C::GFP in worms exposed to t *S. oralis* (VGS#3), *S. mitis* (VGS#10) and *E. coli* OP50.for 2 hours. The degree of nuclear localization of SKN-1B/C::GFP and the percentage of worms in each category fed on *S. oralis* (VGS#3), *S. mitis* (VGS#10) and *E. coli* OP50. A total of more than 100 worms exposed to each strain were imaged and the experiment was repeated 3 times. Significantly high levels of nuclear localization of SKN-1B/C::GFP were observed in worms on *S. oralis* (VGS#3) and *S. mitis* (VGS#10) strains ($P<0.0001$) compared to *E. coli* OP50.
